# Supplementary material for: Cortical connectivity, local dynamics and stability correlates of global conscious states
Source: Commun Biol. 2025 Sep 30;8:1391. doi: 10.1038/s42003-025-08782-6 (PMC12485199; doi:10.1038/s42003-025-08782-6)
Supplement: Supplementary file 2 — Supplementary Information pdf [file 42003_2025_8782_MOESM2_ESM.pdf]

Cortical connectivity, local dynamics and stability correlates of global conscious states

Yun Zhao[1,2,3], Naotsugu Tsuchiya[4,5,6], Mario Boley[1], Vidushani Dhanawansa[1], Yueyang Liu[1], Philippa J. Karoly[7], Andria Pelentritou[8], William Woods[9], David Liley[9,10] and Levin Kuhlmann[1*]

[1]Department of Data Science and Artificial Intelligence, Faculty of Information Technology, Monash University, Clayton, VIC, Australia.
[2]Brain and Mind Centre, The University of Sydney, Camperdown, NSW, Australia.
[3]School of Health Sciences, The University of Sydney, Camperdown, NSW, Australia.
[4]School of Psychological Sciences and Turner Institute for Brain and Mental Health, Monash University, Clayton, VIC, Australia.
[5]Center for Information and Neural Networks (CiNet), National Institute of Information and Communications Technology (NICT), Suita, Japan.
[6]Advanced Telecommunications Research Computational Neuroscience Laboratories, Kyoto, Japan.
[7]Department of Biomedical Engineering, The University of Melbourne, Victoria, Australia.
[8]Laboratoire de Recherche en Neuroimagerie (LREN), University Hospital (CHUV) and University of Lausanne (UNIL), Lausanne, Switzerland.
[9]School of Health Sciences, Swinburne University of Technology, Hawthorn, VIC, Australia.
[10]Department of Medicine-St Vincent's Hospital, The University of Melbourne, Parkville, VIC, Australia.

*Corresponding author(s). E-mail(s): levin.kuhlmann@monash.edu;

Supplementary Note

Definition of matrices A, B, and C in neural mass model

The structure of the matrix $\mathbf{A}$ is composed of independent diagonal blocks, arranged as follows

$$\mathbf{A} = \begin{bmatrix} \mathbf{\Psi} & \mathbf{0} \\ \mathbf{0} & \mathbf{I}_{n_\theta, n_\theta} \end{bmatrix}. \tag{1}$$

Here, $\mathbf{I}_{n_\theta, n_\theta} \in \mathbb{R}^{n_\theta \times n_\theta}$ represents the identity matrix, where n_θ corresponds to the total number of parameters within the system. The component $\mathbf{\Psi} \in \mathbb{R}^{n_x \times n_x}$ is another diagonal matrix formed from smaller sub-blocks, with n_x denoting the state count

$$\mathbf{\Psi} = \mathrm{diag}(\mathbf{\Psi}_j). \tag{2}$$

Each sub-matrix $\mathbf{\Psi}_j$ in $\mathbf{\Psi}$ is expressed as

$$\mathbf{\Psi}_j = \begin{bmatrix} 0 & 1 \\ -\frac{1}{\tau_j^2} & -\frac{2}{\tau_j} \end{bmatrix}, \tag{3}$$

where j iterates over the range $1, 2, \ldots, N$, representing different connections.

For a discrete representation, $\mathbf{A}_\delta$ is derived from $\mathbf{A}$ as follows

$$\mathbf{A}_\delta = \begin{bmatrix} \mathbf{I} + \delta\mathbf{\Psi} & \mathbf{0} \\ \mathbf{0} & \mathbf{I} \end{bmatrix}. \tag{4}$$

The matrix $\mathbf{B}$ is configured with the layout

$$\mathbf{B} = \begin{bmatrix} \mathbf{0}_{n_x, n_x} & \vartheta \\ \mathbf{0}_{n_\theta, n_x} & \mathbf{0}_{n_\theta, n_\theta} \end{bmatrix}. \tag{5}$$

The term $\vartheta \in \mathbb{R}^{n_x \times n_\theta}$ serves as a mapping operator connecting relevant components to a nonlinear activation, defined as

$$\vartheta = \begin{bmatrix} 0 & \ldots & 0 \\ 1 & & 0 \\ \vdots & \ddots & \vdots \\ 0 & & 0 \\ 0 & \ldots & 1 \end{bmatrix}. \tag{6}$$

The discrete variant of $\mathbf{B}$ is expressed by

$$\mathbf{B}_\delta = \delta\mathbf{B}. \tag{7}$$

The connection matrix $\mathbf{C}$ remains identical for both continuous and discrete formulations, structured as

$$\mathbf{C} = \mathrm{diag}(\mathbf{\Pi}, \mathbf{0}_{n_\theta, n_\theta}). \tag{8}$$

The block $\mathbf{\Pi} \in \mathbb{R}^{n_x \times n_x}$ is actually an adjacency matrix (i.e., containing zeros and ones) defining the structure of local connections and consolidates incoming signals to compute mean states, subsequently passing them through an activation function. Its specific

configuration is

$$\mathbf{\Pi} = \begin{bmatrix} 0 & 0 & \dots & 0 & 0 \\ c_{2,1} & 0 & & c_{2,n_x-1} & 0 \\ \vdots & & \ddots & & \vdots \\ 0 & 0 & & 0 & 0 \\ c_{n_x,1} & 0 & & c_{n_x,n_x-1} & 0 \end{bmatrix}. \quad (9)$$

For more detailed description of the neural mass model and its mathematical implementation, readers are encouraged to see [1].

Supplementary Tables

Network	Brain structure	Network	Brain structure	Network	Brain structure
DMN	Parietal_Sup_L	*ECN*	Frontal_Sup_L	*SMN*	Precentral_L
	Parietal_Sup_R		Frontal_Sup_R		Precentral_R
	Parietal_Inf_L		Frontal_Sup_Orb_L		Frontal_Mid_L
	Parietal_Inf_R		Frontal_Sup_Orb_R		Frontal_Mid_R
	Temporal_Mid_L		Frontal_Mid_L		Supp_Motor_Area_L
	Temporal_Mid_R		Frontal_Mid_R		Supp_Motor_Area_R
			Frontal_Mid_Orb_L		Postcentral_L
DMNa	Insula_L		Frontal_Mid_Orb_R		Postcentral_R
	Insula_R		Frontal_Inf_Oper_L		SupraMarginal_L
	Cingulum_Ant_L		Frontal_Inf_Oper_R		SupraMarginal_R
	Cingulum_Ant_R		Frontal_Inf_Tri_L		Paracentral_Lobule_L
	Temporal_Pole_Mid_L		Frontal_Inf_Tri_R		Paracentral_Lobule_R
	Temporal_Pole_Mid_R		Frontal_Inf_Orb_L		
			Frontal_Inf_Orb_R	*VN*	Calcarine_L
DMNv	Cingulum_Post_L		Frontal_Sup_Medial_L		Calcarine_R
	Cingulum_Post_R		Frontal_Sup_Medial_R		Cuneus_L
	Hippocampus_L		Frontal_Med_Orb_L		Cuneus_R
	Hippocampus_R		Frontal_Med_Orb_R		Lingual_L
	ParaHippocampal_L		Rectus_L		Lingual_R
	ParaHippocampal_R		Rectus_R		Occipital_Sup_L
	Fusiform_L		Cingulum_Ant_L		Occipital_Sup_R
	Fusiform_R		Cingulum_Ant_R		Occipital_Mid_L
	Angular_L		Postcentral_L		Occipital_Mid_R
	Angular_R		Postcentral_R		Occipital_Inf_L
	Precuneus_L		Parietal_Sup_L		Occipital_Inf_R
	Precuneus_R		Parietal_Sup_R		
			Parietal_Inf_L		
AN	Insula_L		Parietal_Inf_R		
	Insula_R		SupraMarginal_L		
	Heschl_L		SupraMarginal_R		
	Heschl_R		Angular_L		
	Temporal_Sup_L		Angular_R		
	Temporal_Sup_R		Precuneus_L		
			Precuneus_R		

Table S1 Brain structures in functional networks are shown in the format of Automated Anatomical Labeling atlas [2]. The specific brain structures are identified based on previous studies [3–5]. Seven functional networks: Default Mode Network (DMN), anterior Default Mode Network (DMNa), ventral Default Mode Network (DMNv), Auditory Network (AN), Executive Control Network (ECN), Sensorimotor Network (SMN), Visual Network (VN).

Network	Brain structure	Network	Brain structure
Prefrontal Network	Frontal_Sup_L	*Posterior Parietal Network*	Postcentral_L
	Frontal_Sup_R		Postcentral_R
	Frontal_Sup_Orb_L		Parietal_Sup_L
	Frontal_Sup_Orb_R		Parietal_Sup_R
	Frontal_Mid_L		Parietal_Inf_L
	Frontal_Mid_R		Parietal_Inf_R
	Frontal_Mid_Orb_L		SupraMarginal_L
	Frontal_Mid_Orb_R		SupraMarginal_R
	Frontal_Inf_Oper_L		Angular_L
	Frontal_Inf_Oper_R		Angular_R
	Frontal_Inf_Tri_L		Precuneus_L
	Frontal_Inf_Tri_R		Precuneus_R
	Frontal_Inf_Orb_L		
	Frontal_Inf_Orb_R		
	Frontal_Sup_Medial_L		
	Frontal_Sup_Medial_R		
	Frontal_Med_Orb_L		
	Frontal_Med_Orb_R		
	Rectus_L		
	Rectus_R		

Table S2 Brain structures in prefrontal network and posterior parietal network in the format of Automated Anatomical Labeling atlas. The specific brain structures are identified based on the previous study [6].

Network	Brain structure	Correlation Imaging α_{pi}	Correlation Imaging α_{pe}	Correlation Imaging α_{ep}	Correlation Imaging μ	Contrast Imaging α_{pi}	Contrast Imaging α_{pe}	Contrast Imaging α_{ep}	Contrast Imaging μ
DMN	Parietal_Sup_L			3.9389	3.7324			-8.4166	
	Parietal_Sup_R			3.4843					
	Parietal_Inf_L								
	Parietal_Inf_R			3.4857					
	Temporal_Mid_L								
	Temporal_Mid_R								-3.4651
DMNa	Insula_L								
	Insula_R								-3.3862
	Cingulum_Ant_L						-4.1818		
	Cingulum_Ant_R								
	Temporal_Pole_Mid_L	3.7224	3.7168			-4.0599			
	Temporal_Pole_Mid_R						-4.8823		
DMNv	Cingulum_Post_L		4.0126				-4.1181		
	Cingulum_Post_R			3.6354					
	Hippocampus_L								
	Hippocampus_R								
	ParaHippocampal_L								-3.4271
	ParaHippocampal_R								-4.2258
	Fusiform_L								
	Fusiform_R						-4.6501		
	Angular_L			3.7478	3.5735			-9.3609	
	Angular_R								
	Precuneus_L			3.2482			-3.904		
	Precuneus_R		3.4036	3.5353					
SMN	Precentral_L								
	Precentral_R								
	Frontal_Mid_L								
	Frontal_Mid_R								
	Supp_Motor_Area_L								
	Supp_Motor_Area_R								
	Postcentral_L								
	Postcentral_R								
	SupraMarginal_L								
	SupraMarginal_R				3.6124				
	Paracentral_Lobule_L								
	Paracentral_Lobule_R			3.2410					
VN	Calcarine_L			3.2163				-7.0919	
	Calcarine_R		3.4504	3.8012	3.5762			-7.3398	
	Cuneus_L		3.5177	3.4961	3.3911			-9.2590	-3.7809
	Cuneus_R			3.5109					
	Lingual_L			3.9112	4.1726			-7.1325	-3.8016
	Lingual_R		3.8917						-3.4081
	Occipital_Sup_L		3.8459					-7.0552	
	Occipital_Sup_R			3.7282					
	Occipital_Mid_L			4.0056					
	Occipital_Mid_R							-9.0837	
	Occipital_Inf_L			3.5362				-7.8248	
	Occipital_Inf_R			3.3933				-8.7592	
AN	Insula_L								
	Insula_R								-3.3862
	Heschl_L								-3.4770
	Heschl_R								
	Temporal_Sup_L								
	Temporal_Sup_R								
ECN	Frontal_Sup_L								-3.4626
	Frontal_Sup_R								-3.9891
	Frontal_Sup_Orb_L					-4.7673			
	Frontal_Sup_Orb_R								
	Frontal_Mid_L								
	Frontal_Mid_R								
	Frontal_Mid_Orb_L	4.2717				-4.7284			
	Frontal_Mid_Orb_R								
	Frontal_Inf_Oper_L								-4.3361
	Frontal_Inf_Oper_R								
	Frontal_Inf_Tri_L								
	Frontal_Inf_Tri_R								
	Frontal_Inf_Orb_L					-3.7773			
	Frontal_Inf_Orb_R								
	Frontal_Sup_Medial_L								
	Frontal_Sup_Medial_R								-3.5087
	Frontal_Med_Orb_L		3.4132			-4.1911			
	Frontal_Med_Orb_R		3.4348						
	Rectus_L						-5.6424		
	Rectus_R								
	Cingulum_Ant_L						-4.1818		
	Cingulum_Ant_R								
	Postcentral_L								
	Postcentral_R								
	Parietal_Sup_L			3.9389	3.7324			-8.4166	
	Parietal_Sup_R			3.4843					
	Parietal_Inf_L								
	Parietal_Inf_R			3.4857					
	SupraMarginal_L								
	SupraMarginal_R				3.6124				
	Angular_L			3.7478	3.5735			-9.3609	
	Angular_R								
	Precuneus_L			3.2482			-3.9040		
	Precuneus_R		3.4036	3.5353					

Table S3 Functional networks, and the corresponding brain structures and group-level t-statistics for regional neurophysiological variables in correlation imaging and contrast imaging. Multiple comparisons permutation tests were used (see "Methods") [7].

Supplementary Figures

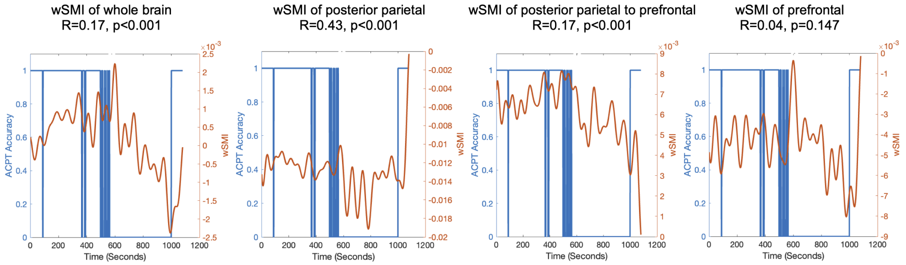

Fig. S1 Time-resolved relationship between behavioural responsiveness and electrophysiological information sharing during Xenon sedation. Blue traces depict Auditory Choice Performance Task (ACPT) accuracy (left y-axis) and orange traces show weighted Symbolic Mutual Information (wSMI; right y-axis) extracted from magnetoencephalography (MEG) in a representative participant. wSMI was computed in brain networks for the (left-to-right) whole brain, posterior parietal cortex, posterior parietal to prefrontal cortex, and prefrontal cortex. In each panel the Pearson correlation between ACPT accuracy and the corresponding wSMI time-series is indicated in the title.

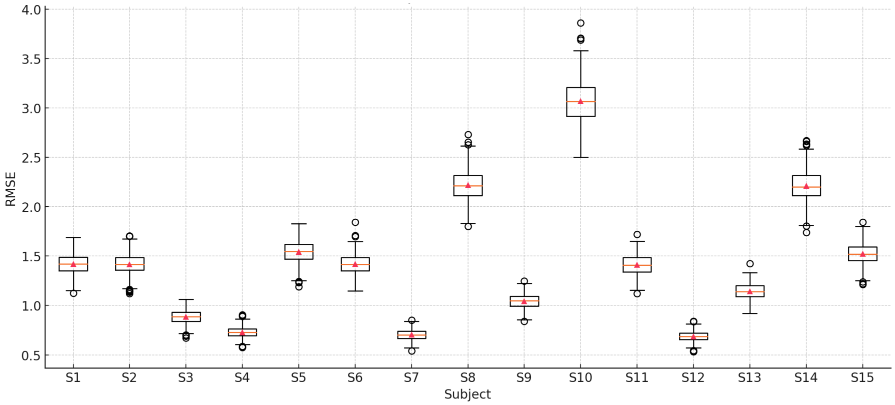

Fig. S2 Root mean squared error between measured and predicted MEG signals for all subjects. Box plots show median (red bar), 1st and 3rd quartiles of the RMSE values for each subject. Median of the RMSE distributions ranged from 0.7 to 3.1, and the amplitude of the measured MEG signal ranges from approximately 15 to 32, reflecting errors below 10% for real data.

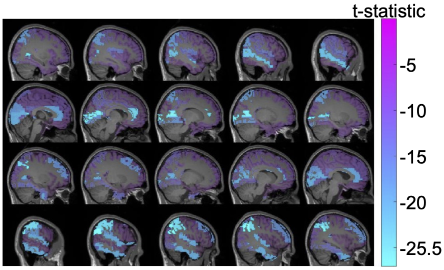

Fig. S3 The cortical regions where the membrane potential of the pyramidal population exhibited significant differences before and after Xenon equilibrated are presented. Significant regions were identified by t-statistics derived from the multiple comparisons permutation test at the significance level $\alpha = 0.05$.

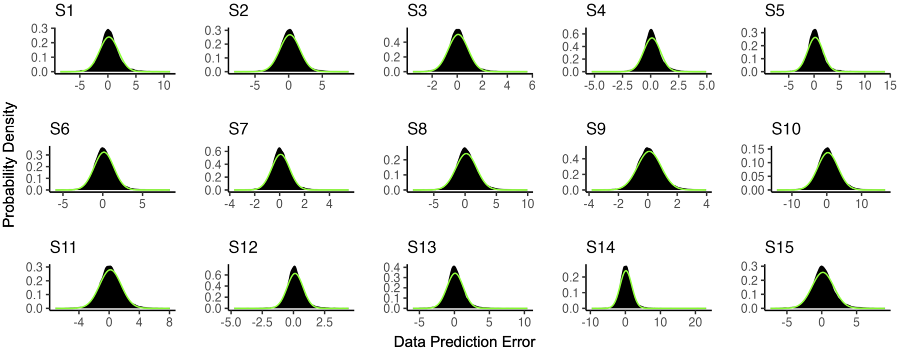

Fig. S4 Neural mass models were forward simulated using parameter estimates. The data prediction error is the difference between the source-level MEG time series and the forward simulation. The data prediction errors for all 15 subjects are depicted as probability density functions (black histograms), with normal probability density functions having equivalent mean and standard deviation (green curves).

References

[1] Freestone, D. R. *et al.* Estimation of effective connectivity via data-driven neural modeling. *Frontiers in neuroscience* 383 (2014) .

[2] Tzourio-Mazoyer, N. *et al.* Automated anatomical labeling of activations in spm using a macroscopic anatomical parcellation of the mni mri single-subject brain. *Neuroimage* **15** (1), 273–289 (2002) .

[3] Farras-Permanyer, L. *et al.* Age-related changes in resting-state functional connectivity in older adults. *Neural regeneration research* **14** (9), 1544–1555 (2019) .

[4] Brookes, M. J. *et al.* Investigating the electrophysiological basis of resting state networks using magnetoencephalography. *Proceedings of the National Academy of Sciences* **108** (40), 16783–16788 (2011) .

[5] Beckmann, C. F., DeLuca, M., Devlin, J. T. & Smith, S. M. Investigations into resting-state connectivity using independent component analysis. *Philosophical Transactions of the Royal Society B: Biological Sciences* **360** (1457), 1001–1013 (2005) .

[6] Boly, M. *et al.* Are the neural correlates of consciousness in the front or in the back of the cerebral cortex? clinical and neuroimaging evidence. *Journal of Neuroscience* **37** (40), 9603–9613 (2017) .

[7] Nichols, T. E. & Holmes, A. P. Nonparametric permutation tests for functional neuroimaging: a primer with examples. *Human brain mapping* **15** (1), 1–25 (2002) .
