# Supplementary material for: Cortical connectivity, local dynamics and stability correlates of global conscious states
Source: Commun Biol. 2025 Sep 30;8:1391. doi: 10.1038/s42003-025-08782-6 (PMC12485199; doi:10.1038/s42003-025-08782-6)
Supplement: Supplementary file 3 — Reporting summary [file 42003_2025_8782_MOESM3_ESM.pdf]

nature portfolio

Corresponding author(s): COMMSBIO-25-0106A
Last updated by author(s): 2025-6-15

Reporting Summary

Nature Portfolio wishes to improve the reproducibility of the work that we publish. This form provides structure for consistency and transparency in reporting. For further information on Nature Portfolio policies, see our Editorial Policies and the Editorial Policy Checklist.

Statistics

For all statistical analyses, confirm that the following items are present in the figure legend, table legend, main text, or Methods section.

n/a	Confirmed	
☐	☒	The exact sample size (*n*) for each experimental group/condition, given as a discrete number and unit of measurement
☐	☒	A statement on whether measurements were taken from distinct samples or whether the same sample was measured repeatedly
☐	☒	The statistical test(s) used AND whether they are one- or two-sided<br>*Only common tests should be described solely by name; describe more complex techniques in the Methods section.*
☒	☐	A description of all covariates tested
☐	☒	A description of any assumptions or corrections, such as tests of normality and adjustment for multiple comparisons
☐	☒	A full description of the statistical parameters including central tendency (e.g. means) or other basic estimates (e.g. regression coefficient) AND variation (e.g. standard deviation) or associated estimates of uncertainty (e.g. confidence intervals)
☐	☒	For null hypothesis testing, the test statistic (e.g. *F*, *t*, *r*) with confidence intervals, effect sizes, degrees of freedom and *P* value noted<br>*Give P values as exact values whenever suitable.*
☒	☐	For Bayesian analysis, information on the choice of priors and Markov chain Monte Carlo settings
☐	☒	For hierarchical and complex designs, identification of the appropriate level for tests and full reporting of outcomes
☒	☐	Estimates of effect sizes (e.g. Cohen's *d*, Pearson's *r*), indicating how they were calculated

Our web collection on statistics for biologists contains articles on many of the points above.

Software and code

Policy information about availability of computer code

Data collection	This study used data collected from a previous research that investigated changes in EEG/MEG signals from 15 subjects during Xenon-anaesthetic-induced reductions of consciousness. See "Recording brain electromagnetic activity during the administration of the gaseous anesthetic agents xenon and nitrous oxide in healthy volunteers" for details.
Data analysis	This study used customized MATLAB code to process and analyze the data. The code can be found on GitHub: https://github.com/yundumbledore/NeuroProcImager-Pro.

For manuscripts utilizing custom algorithms or software that are central to the research but not yet described in published literature, software must be made available to editors and reviewers. We strongly encourage code deposition in a community repository (e.g. GitHub). See the Nature Portfolio guidelines for submitting code & software for further information.

Data

Policy information about availability of data

All manuscripts must include a data availability statement. This statement should provide the following information, where applicable:

- Accession codes, unique identifiers, or web links for publicly available datasets
- A description of any restrictions on data availability
- For clinical datasets or third party data, please ensure that the statement adheres to our policy

The source-level MEG data will be made publicly accessible upon the publication of the manuscript.

Research involving human participants, their data, or biological material

Policy information about studies with human participants or human data. See also policy information about sex, gender (identity/presentation), and sexual orientation and race, ethnicity and racism.

Reporting on sex and gender	Male participants were recruited for the study. Exclude females due to the documented effects of menstruation and/or age extremes on the resting MEG/EEG signal as well as the increased propensity to nausea and vomiting.
Reporting on race, ethnicity, or other socially relevant groupings	This research does not involve any considerations related to race, ethnicity, or other socially relevant groupings. The study design, data collection, and analysis are independent of such classifications and do not incorporate these variables in any form.
Population characteristics	The participants are all between 20 and 40 years old. They confirm a good general health status by obtaining the participant's Body Mass Index (BMI) and the lack of contraindications to MRI or MEG (such as implanted metallic foreign bodies), as well as collecting a detailed medical history including any previous surgeries, importantly any unfavorable reactions to general anesthesia which would result in exclusion from the study. The team who collected the data specifically excluded any recent intake of psychoactive or other prescribed medication as well as ensuring the absence of any recreational drug use and the lack of any neurological disorder, epilepsy, heart conditions, sleep apnea, motion sickness and claustrophobia.
Recruitment	This study does not involve active recruitment of human participants, their data, or biological material. As such, no recruitment strategies, processes, or materials were utilized in the scope of this research.
Ethics oversight	Alfred Hospital, Melbourne, Victoria, Australia

Note that full information on the approval of the study protocol must also be provided in the manuscript.

Field-specific reporting

Please select the one below that is the best fit for your research. If you are not sure, read the appropriate sections before making your selection.

☒ Life sciences ☐ Behavioural & social sciences ☐ Ecological, evolutionary & environmental sciences

For a reference copy of the document with all sections, see nature.com/documents/nr-reporting-summary-flat.pdf

Life sciences study design

All studies must disclose on these points even when the disclosure is negative.

Sample size	The sample size of 15 subjects was chosen based on the specific objectives and scope of this study. This number was determined to be sufficient for obtaining reliable and meaningful insights while considering practical constraints such as resource availability, time, and ethical considerations. The study focuses on detailed, in-depth analysis rather than large-scale generalizability, making a smaller sample size appropriate. Furthermore, previous similar studies in the field have successfully utilized comparable sample sizes to achieve robust results. Additionally, the statistical power was assessed to ensure that the sample size would allow for meaningful interpretation of the data without compromising validity.
Data exclusions	No data were excluded.
Replication	All attempts at replication were successful.
Randomization	It is not relevant to our study because our study does not involve multiple experimental groups.
Blinding	It is not relevant to this study because this study used data that were collected by a previous research.

Reporting for specific materials, systems and methods

We require information from authors about some types of materials, experimental systems and methods used in many studies. Here, indicate whether each material, system or method listed is relevant to your study. If you are not sure if a list item applies to your research, read the appropriate section before selecting a response.

Materials & experimental systems

n/a	Involved in the study
☒	☐ Antibodies
☒	☐ Eukaryotic cell lines
☒	☐ Palaeontology and archaeology
☒	☐ Animals and other organisms
☒	☐ Clinical data
☒	☐ Dual use research of concern
☒	☐ Plants

Methods

n/a	Involved in the study
☒	☐ ChIP-seq
☒	☐ Flow cytometry
☒	☐ MRI-based neuroimaging

Plants

Seed stocks	This is not relevant to this study.
Novel plant genotypes	This is not relevant to this study.
Authentication	This is not relevant to this study.
